# EggCountAI: a convolutional neural network-based software for counting of *Aedes aegypti* mosquito eggs

**DOI:** 10.1186/s13071-023-05956-1

**Published:** 2023-10-02

**Authors:** Nouman Javed, Adam J. López-Denman, Prasad N. Paradkar, Asim Bhatti

**Affiliations:** 1 Institute for Intelligent Systems Research and Innovation, Deakin University, Geelong, VIC 3216 Australia; 2 CSIRO Health & Biosecurity, Australian Centre for Disease Preparedness, Geelong, VIC 3220 Australia

**Keywords:** Fecundity, Artificial intelligence, Mask RCNN, Egg counting, EggCountAI, ICount, MECVision, Epidemiological models

## Abstract

**Background:**

Mosquito-borne diseases exert a huge impact on both animal and human populations, posing substantial health risks. The behavioural and fitness traits of mosquitoes, such as locomotion and fecundity, are crucial factors that influence the spread of diseases. In existing egg-counting tools, each image requires separate processing with adjustments to various parameters such as intensity threshold and egg area size. Furthermore, accuracy decreases significantly when dealing with clustered or overlapping eggs. To overcome these issues, we have developed EggCountAI, a Mask Region-based Convolutional Neural Network (RCNN)-based free automatic egg-counting tool for *Aedes aegypti* mosquitoes.

**Methods:**

The study design involves developing EggCountAI for counting mosquito eggs and comparing its performance with two commonly employed tools—ICount and MECVision—using 10 microscopic and 10 macroscopic images of eggs laid by females on a paper strip. The results were validated through manual egg counting on the strips using ImageJ software. Two different models were trained on macroscopic and microscopic images to enhance egg detection accuracy, achieving mean average precision, mean average recall, and F1-scores of 0.92, 0.90, and 0.91 for the microscopic model, and 0.91, 0.90, and 0.90 for the macroscopic model, respectively. EggCountAI automatically counts eggs in a folder containing egg strip images, offering adaptable filtration for handling impurities of varying sizes.

**Results:**

The results obtained from EggCountAI highlight its remarkable performance, achieving overall accuracy of 98.88% for micro images and 96.06% for macro images. EggCountAI significantly outperformed ICount and MECVision, with ICount achieving 81.71% accuracy for micro images and 82.22% for macro images, while MECVision achieved 68.01% accuracy for micro images and 51.71% for macro images. EggCountAI also excelled in other statistical parameters, with mean absolute error of 1.90 eggs for micro, 74.30 eggs for macro, and a strong correlation and *R*-squared value (0.99) for both micro and macro. The superior performance of EggCountAI was most evident when handling overlapping or clustered eggs.

**Conclusion:**

Accurate detection and counting of mosquito eggs enables the identification of preferred egg-laying sites and facilitates optimal placement of oviposition traps, enhancing targeted vector control efforts and disease transmission prevention. In future research, the tool holds the potential to extend its application to monitor mosquito feeding preferences.

**Graphical Abstract:**

**Figure:**
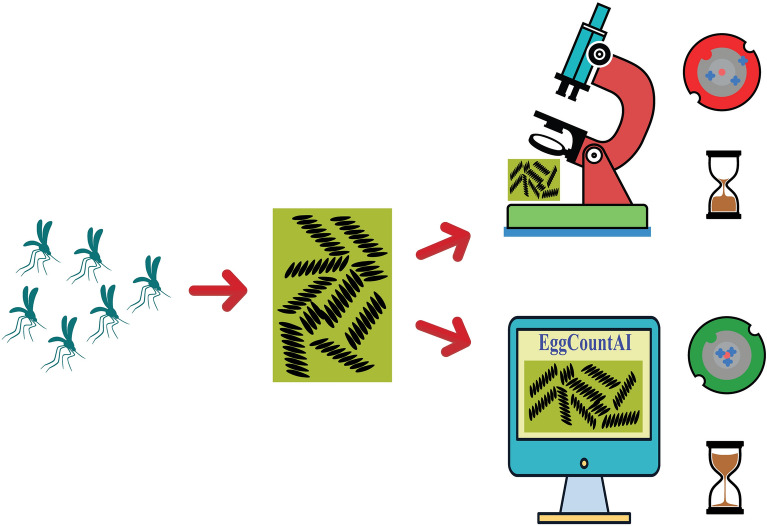

## Background

Mosquito-transmitted pathogens are considered deadly due to their worldwide impact on public health [1]. Annually, they are responsible for around 230 million cases of malaria [2], significantly impacting children under 5 years of age [3], while the dengue virus causes an estimated 390 million infections, with about 20,000 deaths per year [4]. Despite over 100 years of research and deployment of mosquito control approaches including long-lasting insecticidal nets (LLINs) and indoor residual spraying (IRS), diseases transmitted by mosquitoes remain a concern worldwide [5]. The impact of mosquito-transmitted diseases will also likely worsen in the future due to the increase in key factors of pathogen spread, such as the growing population, urbanisation, lack of infrastructure in large cities, climate change, insecticide resistance, travel, and trade [6].

Infections with pathogens can modulate different behavioural and fitness traits of mosquitoes such as locomotion, blood-feeding, fecundity, and fertility [7]. Mosquito-borne viruses can also significantly impact the mosquito’s nervous system, leading to behavioural changes that influence their transmission of mosquito-borne diseases [8–10]. In an epidemiological context, it is necessary to assess mosquito fitness, as this may aid in understanding how different mosquito populations adapt to environmental conditions [11, 12] and the interactions between vectors and their pathogens [13, 14]. Fecundity, the number of eggs laid by female mosquitoes, is a critical trait in determining a mosquito’s fitness, and has been used as a marker to assess the population fitness of mosquitoes [15]. Fecundity also helps in estimating the population density and infestation of the vector in a specific area by assessing the number of eggs present in the designated area, on a spatio-temporal basis, considering both spatial distribution and changes over time [16].

*Aedes aegypti* mosquitos are the primary vector of several medically significant viruses, including dengue, Zika, yellow fever, and chikungunya. Female mosquitoes can lay anywhere from 20 to 140 eggs per blood meal depending on factors such as the quantity of blood consumed and the female's body size and reproductive capacity [17]. Additionally, the *Aedes* mosquito’s reproductive success is influenced by its eco-biological features, including skip oviposition, where females lay eggs in multiple batches in different breeding sites for diverse offspring distribution [18]. The ability of their eggs to enter a dormant state (quiescence) during unfavourable conditions ensures survival and widespread dispersion [19]. With both active (larvae, pupae, adults) and passive (quiescent eggs) populations present, *Aedes* mosquitoes maintain a continuous presence in the environment, making them significant disease vectors. Understanding these factors is vital for effective vector control and disease prevention strategies. Novel vector control strategies such as the release of modified mosquitoes involve the mass rearing and production of thousands of mosquitoes, possibly over many gonotrophic cycles and generations, requiring the counting of hundreds of thousands of eggs [20]. Additionally, in endemic areas, egg samples can be highly complex, with multiple layers of eggs. Counting the number of eggs manually with the aid of a magnifying lens or optical microscope makes the process tedious, slow, laborious, and prone to errors.

The automatic counting of mosquito eggs is a subject that has received considerable attention in the last few years, with several automatic egg-counting tools having recently been developed. Some of these tools use segmentation to acquire pixels containing mosquito eggs [21, 22], while others provide graphical user interfaces through a web app [23] or computer software [24, 25]. A recent study also employed a wavelet-based method to count eggs [26]; however, unlike the others listed, it requires knowledge of mathematical techniques to process samples. Some tools also required extensive hardware for successful counting, such as scanners, light-emitting diode (LED) lighting, and mechanical support [27, 28]. In most egg-counting tools, although some operations work automatically, each image must be processed separately by manually adjusting the different parameters, such as intensity threshold value and the minimum and maximum size of a unit egg area. Additionally, the performance/accuracy of these current methods significantly decreases when eggs in the image are in clusters or overlap.

Convolutional neural networks (CNNs) are a class of deep neural networks that have revolutionised computer vision tasks by enabling automatic feature extraction [29]. CNNs are equipped with features like instance segmentation and non-maximum suppression (NMS) to accurately identify and segment objects, even when they are overlapping or close together [30]. In mosquito research, CNNs have been used to identify many different characteristics including breeding site detection [31], flight tracking [32], genus classification [33], and egg identification [34]. However, no CNN-based tool has been developed to count the number of eggs laid by mosquitoes. Considering the potential of CNNs, it is hypothesised that the application of CNNs could help in counting mosquito eggs with higher accuracy than current methods.

To overcome limitations such as decreased performance when the eggs in the image are in clusters or overlap, and the tedious requirement of processing each image separately, we have developed a CNN-based free software, EggCountAI, to count *Aedes* mosquito eggs. The software can count eggs laid on a paper strip from a folder containing several micro or macro images, and also offers filtration to remove undesired impurities such as mosquito remains and dust particles. In order to test the software’s capability, we compared EggCountAI with two widely used tools, ICount (developed under the same supervision team) [24] and MECVision (Mosquito Egg Computer Vision) [23].

## Methods

### Maintenance rearing

All experiments were performed under biosafety level 3 (BSL-3) conditions in the insectary at the Australian Centre for Disease Preparedness (ACDP). The *Ae. aegypti* strain used in this study was originally from Cairns, Queensland, and has been kept in the insectary for around 70 generations. This strain was reared at 27.0 °C with 65% relative humidity and a 12-h light/dark cycle. To hatch, previously laid egg strips were introduced to reverse osmosis (RO) water, where impurities and contaminants were eliminated via a semipermeable membrane. Larvae were fed fish food (Sera, Germany) as required until pupae formation, approximately 7 days post hatching. Adult mosquitoes were then transferred to a colony cage and fed 10% sucrose solution ad libitum.

### Artificial blood-feeding and egg collection

Chicken blood meal was provided using chicken skin and an artificial blood-feeding device (Hemotek^®^, Accrington, UK) once a week, lasting 1 h each time. Three days post-blood meal, beakers were deployed in the cage for female oviposition. A strip of sandpaper measuring the perimeter of a 100-ml beaker (160 mm) in length was inserted into the container. The container was then filled with 50 ml of water up to a depth of half the width of the sandpaper strip (25 mm). After the completion of mosquito oviposition, which typically occurred within 5 days of the blood meal, the sandpaper strip was taken out of the colony cage and allowed to dry at room temperature.

### Capturing and processing of images

Once the sandpaper strip containing eggs was collected and dried, images of the strip could be taken at any time. Two distinct types of images were collected, labelled “micro” or “macro”, following a similar pattern as described in other studies [23, 24]. Using a camera (Olympus Tough TG-6), an image of the entire sandpaper strip was captured with the focus on the plane of the eggs. This collection of images will be referred to as “macro” in this paper. The second image type was taken utilising a microscope (Nikon SMZ18) set at 8.0× magnification with a 1.0× objective lens with the Nikon NIS-Elements software (Nikon, Japan). These images will be referred to as “micro”. Macro images were cropped with the help of the Photos app (Microsoft Windows) to eliminate the area without mosquito eggs. Micro images were ready for analysis without any pre-processing.

### Training and validation

Two different models were trained based on macro and micro images to improve accuracy in egg detection. In the case of the micro image-based model, 100 images were collected, with 70 used for training purposes, 20 used for validation, and 10 used for testing purposes. The dataset used for training contained images with egg counts ranging from 13 to 167, while the validation set comprised images with egg counts between 24 and 182. In the testing phase, the images had object counts ranging from 104 to 215. The model was trained for 20 epochs with 500 steps per epoch, and the detection threshold was set to 70%, resulting in the exclusion of proposals with a confidence level below 0.7 out of 1.0. Training and validation data annotations were created with the help of the MakeSense web tool [35] in the form of a .json file.

In the case of the macro image-based model, 100 images were collected, from which 70 were used for training purposes, 20 for validation, and 10 for testing. The dataset used for training contained images with egg counts ranging from 133 to 624, while the validation set comprised images with egg counts between 76 and 357. In the testing phase, the images had object counts ranging from 1047 to 3658. The macro model was also trained for 20 epochs with 500 steps per epoch, and the detection threshold was 70%.

Although our dataset consisted of only 100 micro and 100 macro images, we ensured its diversity by capturing various types of egg clusters and orientations of eggs to thoroughly train and test our model.

### Programming and computational system

All software development and data processing were performed in a Windows 11 Pro 64-bit environment using the AMD Ryzen 9 5900HX with Radeon graphics (3.30 GHz) processor and 2 × 16 GB small outline dual inline memory module (SO-DIMM) double data rate (DDR)4-3200 random access memory RAM. The graphical user interface of EggCountAI was implemented using Python 3.8, TensorFlow 2.40, and OpenCV 4.6.0.66 software.

### Manual counting

**Fig. 1:**
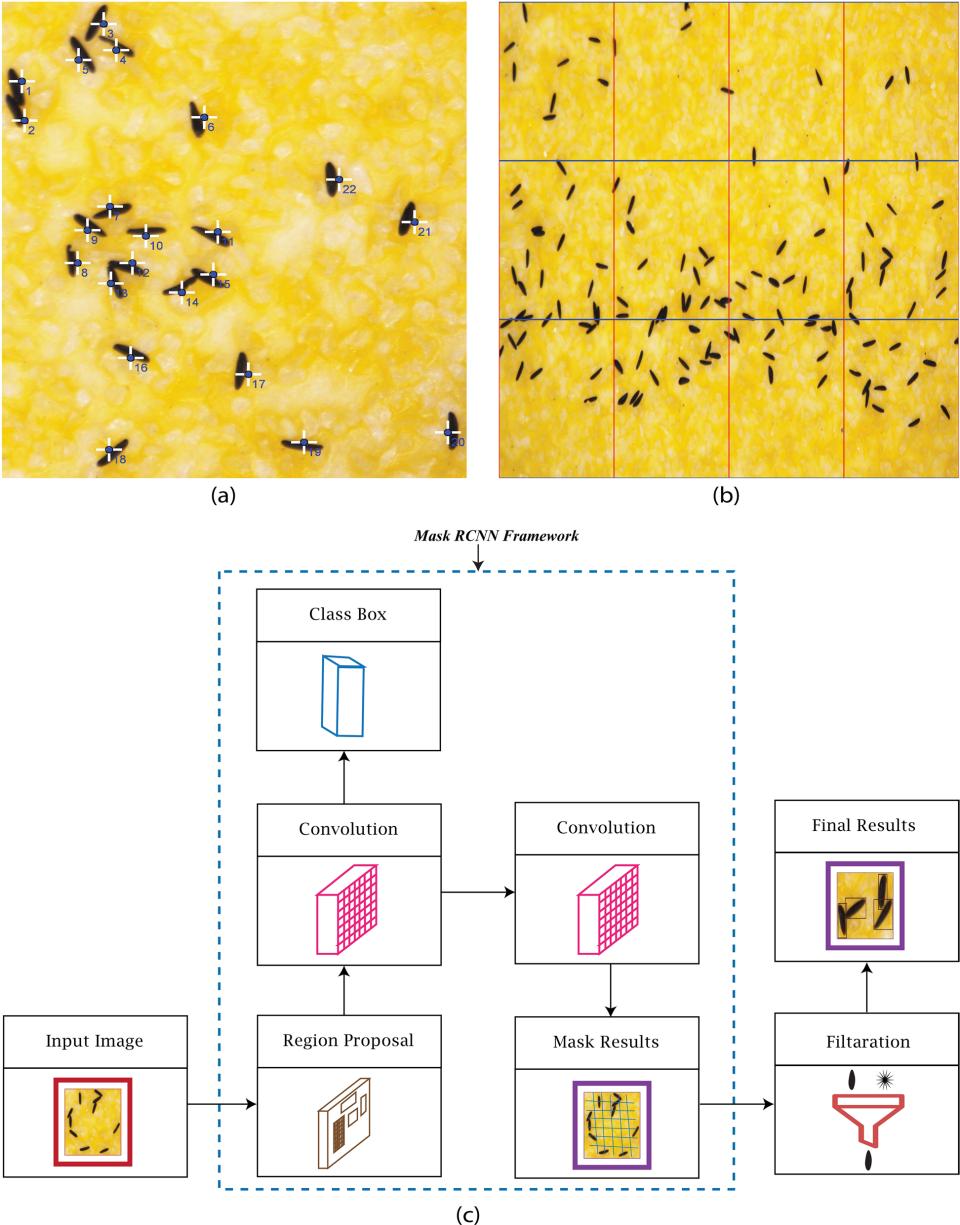
Manual counting, overlying grid on the image, and egg-counting methodology. **a** Example of manual counting using the ImageJ Multi-point Tool. **b** Overlying grid on the image to create patches for processing larger images. The image in the figure shows three horizontal and four vertical divisions, making 12 patches. **c** EggCountAI is based on the Mask RCNN. The input image data feed into the Mask RCNN. Mask RCNN applies its two-stage approach, generates potential object regions, and applies different convolutional and fully connected layers to produce binary masks. The masks that do not fulfil the limits set through the filtration parameter are excluded from the final results

To validate the accuracy of EggCountAI software, a manual egg count was performed using ImageJ software [36]. The Multi-point Tool was selected to insert the multiple points inside the image of the sandpaper strip. The Add [*t*] feature inside the ROI (Region of Interest) Manager of ImageJ helped to add all multi-points along with the decimal count value (Fig. 1a). Decimal count values and Multi-point eliminated the chances of errors in manual counting.

### Performance evaluation and statistical analysis

The performance of tools was validated using percentage accuracy, overall percentage accuracy, mean absolute error, correlation (quantifying the strength of the linear relationship between ground truth egg counts and the egg counts calculated by the tools), and the *R*-squared value. For percentage accuracy, individual images were considered, while for overall percentage accuracy, the same formula was used but all images of the relevant dataset were considered. The formula used for the percentage accuracy is given in Eq. 1.

1 $${\text{Percentage}}\;{\text{accuracy}} = \left( {1 - \frac{{\left| {{\text{number of eggs counted by software}} - {\text{ground truth number of eggs}}} \right|}}{{\text{ground truth number of eggs}}}} \right)*100$$

The SciPy library in Python was used to calculate the mean absolute error, correlation, and *R*-squared value.

### Model architecture

The type of deep neural network used in EggCountAI is the Mask region-based CNN (RCNN). Mask RCNN is a deep learning model that excels in detecting objects and segmenting them in computer vision applications [32]. The architecture of Mask RCNN is a comprehensive composition of several key components, including a backbone network, region proposal network (RPN), RoIAlign layer, bounding box regression and classification head, mask head, loss functions, and training and inference, each playing a vital role in its object detection and segmentation capabilities. At its core is the backbone network, responsible for feature extraction from the input image. In EggCountAI, we utilised ResNet-101 as the backbone network because of its deep architecture and remarkable ability to capture intricate features, resulting in enhanced precision and more detailed segmentation masks. ResNet-101 comprises a total of 101 layers. Each layer is designed to handle distinct tasks, such as edge detection and texture identification. The RPN comes into play next. The RPN processes the input image and generates potential object regions in the form of rectangular bounding boxes. These regions are then refined by the bounding box regression and classification head, which uses fully connected layers to predict class labels and improve the accuracy of bounding box coordinates. For precise feature extraction within proposed regions, the RoIAlign layer is utilised, ensuring optimal alignment without quantisation. The mask head takes these aligned features and predicts binary masks for each detected object instance, enabling precise segmentation. The model training is supported by multiple loss functions, including smooth L1 loss for bounding box regression, cross-entropy loss for classification, and pixel-wise binary cross-entropy loss for mask prediction. The model processes new images during inference, generating object proposals, class predictions, and segmentation masks (Fig. 1c).

### Egg estimation using EggCountAI software

EggCountAI provides five flexible input parameters: (1) choosing between micro and macro image analysis type, (2) setting vertical divisions, (3) setting horizontal divisions, (4) setting the filtration level, and (5) adjusting the detection confidence threshold. Micro and macro options can be selected considering the input data type. Vertical and horizontal divisions can divide the input image into smaller patches, enabling the processing of larger images that would be otherwise infeasible due to memory limitations or computational constraints (Fig. 1b). Both horizontal and vertical divisions accept values from 1 to 40. Setting the value to 1 for both horizontal and vertical divisions means that the input image will not be divided into any number of patches (mainly used for the micro images, as they generally have sufficient zoom). Setting the value to 2 for both means that the input image will be divided into a 2 × 2 grid of four equal-sized patches, and setting the value to 40 for both means that the input image will be divided into a 40 × 40 grid of 1600 equal-sized patches. The division is only for processing purposes, and after processing, the patches will be combined, and the output image will be in the form of the entire input image.

Whilst setting of the horizontal and vertical divisions is mainly for use with macro images, it can also be useful for micro images depending on the magnification of the microscope. The value of horizontal and vertical divisions for this study was two vertical and two horizontal divisions for micro images and 25 vertical and seven horizontal divisions for macro images, as the macro images had greater widths than heights, and the sizes of eggs in the images were also very small. Setting the filtration level parameter can be used to eliminate noise and impurities from the input image. It takes a value within a range from 0.1 to 10.0. Setting the value to 0.1 means that it will eliminate all the objects in each patch which are 0.1 times larger or smaller than the average size of eggs in that patch. Setting the value to 10.0 means that it will eliminate all the objects in each patch which are 10.0 times larger or smaller than the average size of eggs in that patch. The default value of the filtration level is set at 3.0 and kept the same during the analysis in this study. The confidence value shows the minimum confidence level that a detected egg must have to be part of the counted eggs. The confidence value ranges from 0.0 to 1.0, where 1.0 means that the software is fully confident that the detected object is the mosquito egg. A default confidence value of 0.7 was used.

**Fig. 2:**
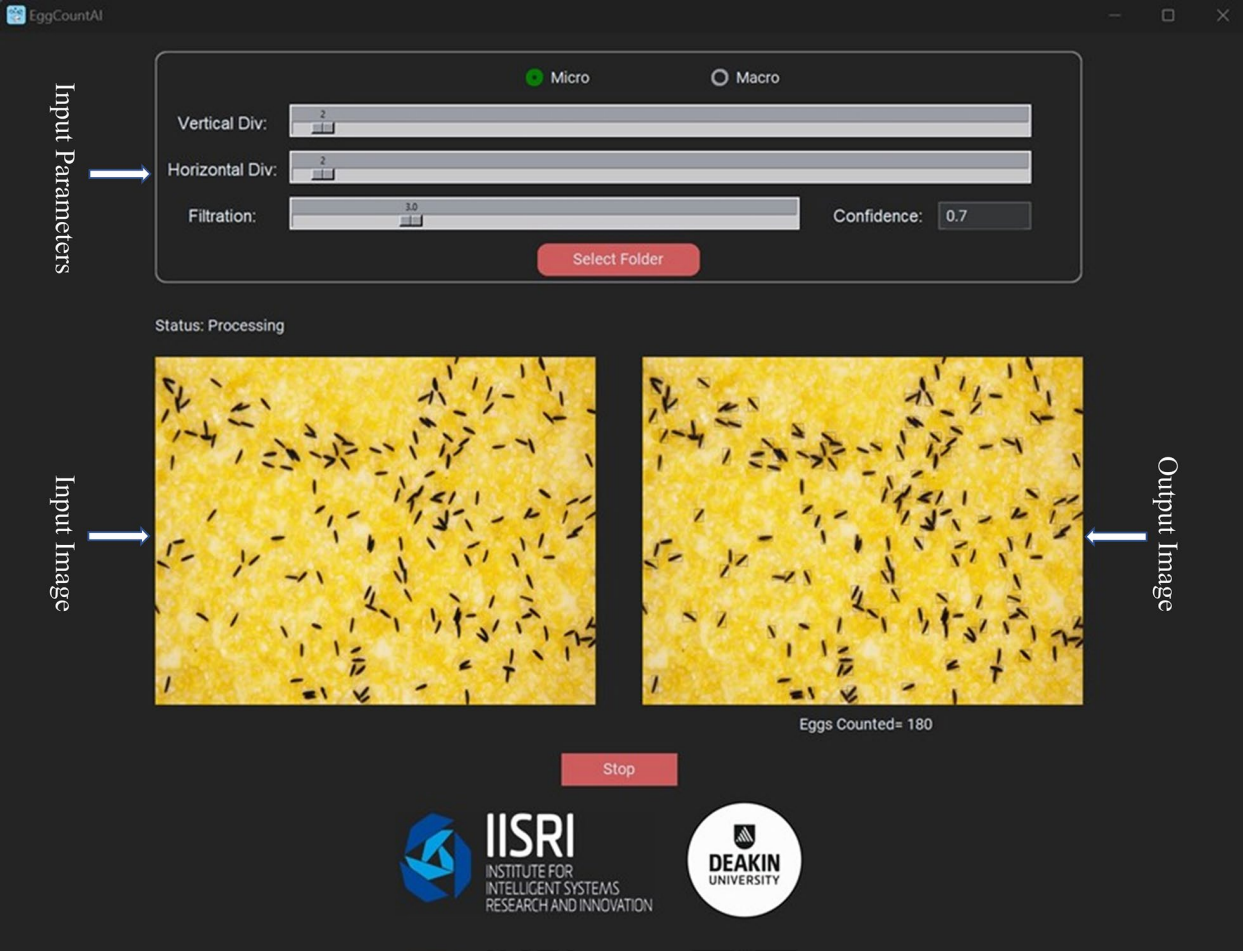
EggCountAI graphical user interface

After these parameters have been set, the input folder containing the macro or micro images can be selected. EggCountAI will create a new folder, named “Results”, inside the input folder during processing that contains the processed input images with a common prefix “Result” in their names. EggCountAI also creates a simple text file in the Results folder containing the names of the processed images and the number of eggs detected. For instance, if the name of the input image is Eggimage1, the Results folder will contain the processed image with the name Result-Eggimage1 and the text file will show the name Result-Eggimage1 along with the number of eggs detected. Besides saving the processed images and logs in the Result folder, the software will show the input images, processed images, and numbers of eggs detected in the user interface. The software also allows the user to stop the process at any time by pressing the stop button and will save the partially processed results. The egg-counting procedure was displayed on the user interface as depicted in Fig. 2.

## Results

**Fig. 3:**
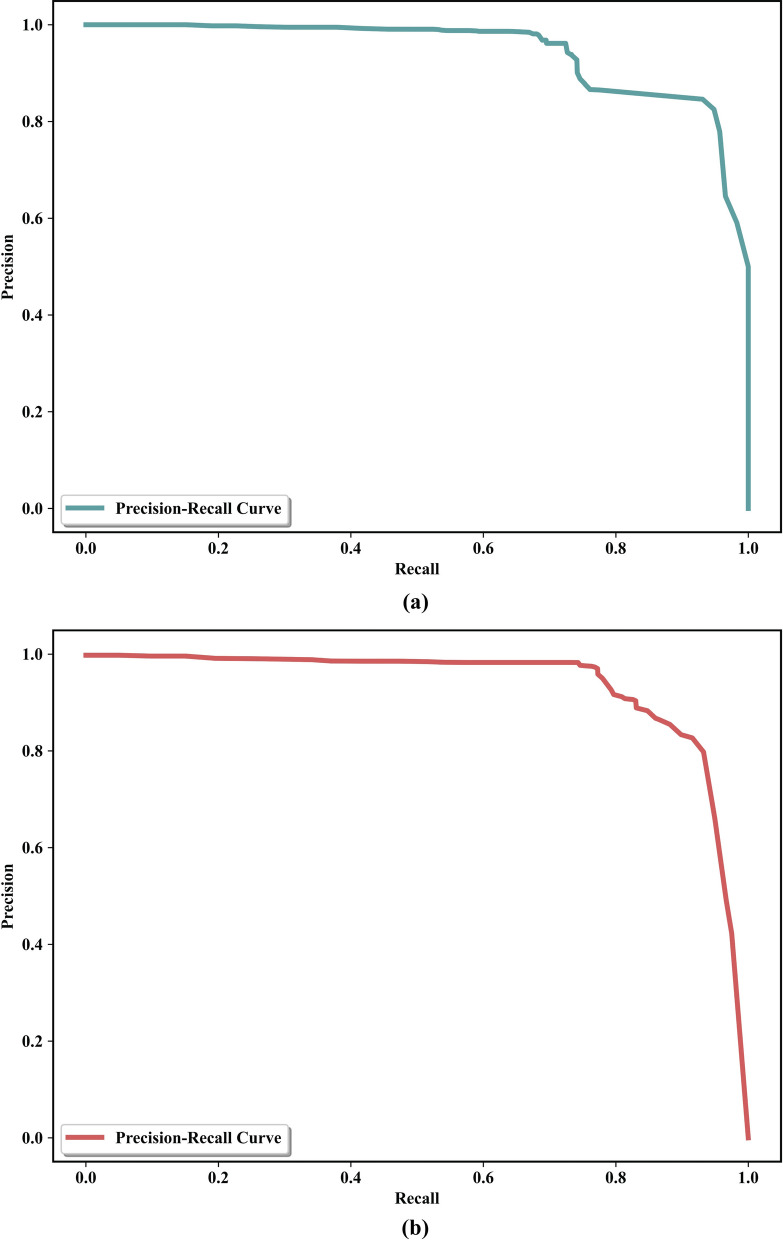
Precision–recall curves. **a** Precision–recall curve based on micro image training. **b** Precision–recall curve based on macro image training

The results obtained from training and validation were impressive, showing mean average precision, mean average recall, and F1-score values of 0.92, 0.90, and 0.91 for the microscopic model and 0.91, 0.90, and 0.90 for the macroscopic model, respectively, despite the limited dataset size. The precision–recall curve for micro image training is given in Fig. 3a, and the curve for macro image training is given in Fig. 3b. The precision–recall curve demonstrates the relationship between precision (positive predictive value) and recall (sensitivity) at various thresholds in binary classification tasks. Although the results were exceptional, we acknowledge the potential for further improvement by incorporating additional strategies for limited datasets, such as data augmentation, to increase the dataset size and improve training artificially. Additionally, the small dataset size may have implications for the model performance and generalizability, and a larger dataset could provide better performance for different scenarios [36]. For instance, studies related to mosquito classification have used more than 4000 images [37, 38], although they dealt with multi-class classification rather than our single-class scenario. Ten macro images and 10 micro images were processed through EggCountAI. Each set of data was processed separately by selecting micro and macro options from the software.

### Micro images

The performance of the EggCountAI tool for micro images was analysed on a dataset of 10 random micro images containing egg numbers ranging from 104 to 215 (Table 1). EggCountAI successfully detected eggs in each image with a high level of accuracy, and was able to identify those that were overlapping, demonstrating the tool’s effectiveness with complex datasets. The high level of accuracy of detection across the datasets analysed showcases EggCountAI’s ability to adapt to different egg patterns, with overall accuracy of 98.88% for the tool across all images.

**Table 1:** EggCountAI results for micro images of *Ae. aegypti* eggs

Image	Ground truth	EggCountAI	Accuracy %
1	189	186	98.41
2	156	153	98.07
3	157	157	100
4	183	180	98.36
5	158	154	97.46
6	177	177	100
7	124	123	99.19
8	215	212	98.60
9	104	104	100
10	150	149	99.33
Total	1613	1595	98.88

**Fig. 4:**
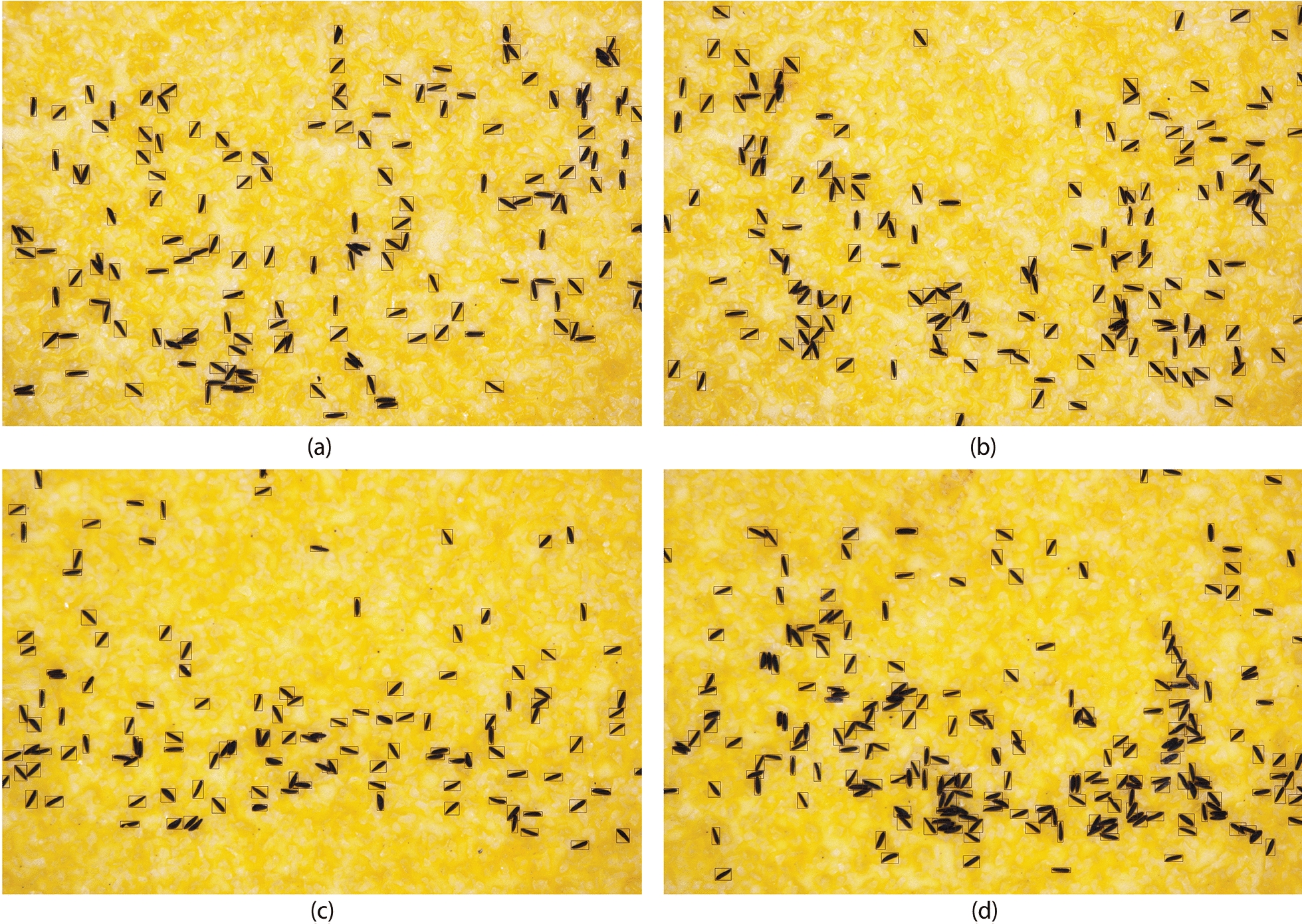
EggCountAI results based on micro images of *Ae. aegypti* eggs, with images showing **a** 153, **b** 157, **c** 123, and **d** 212 eggs detected

To further illustrate the performance of EggCountAI, Fig. 4a–d shows a combined image of four out of the 10 processed images, with rectangles outlining the detected eggs. This visual representation enables a comprehensive understanding of EggCountAI’s performance, highlighting the accurate detection.

### Macro images

The performance of the EggCountAI tool for macro images was analysed on a dataset of 10 random macro images containing between 1047 and 3658 eggs (Table 2). The EggCountAI tool exhibited remarkable efficacy in accurately detecting a significant number of eggs in each image, including instances where the eggs were very close to each other, highlighting its effectiveness in assessing samples obtained without a microscope.

**Table 2:** EggCountAI results for macro images of *Ae. aegypti* eggs

Image	Ground truth	EggCountAI	Accuracy %
1	2377	2185	91.92
2	1579	1544	97.78
3	1821	1775	97.47
4	1416	1391	98.23
5	1490	1439	96.57
6	2126	2112	99.34
7	1748	1724	98.62
8	1047	1041	99.42
9	3658	3344	91.41
10	1611	1575	97.76
Total	18,873	18,130	96.06

Table 2 presents the egg detection results for the macro images. Based on the findings presented in Table 2, it can be observed that EggCountAI achieved above 91% accuracy in all macro images. The overall accuracy was 96.06%, showing that EggCountAI can achieve outstanding results even if macro images are fed with thousands of eggs.

**Fig. 5:**
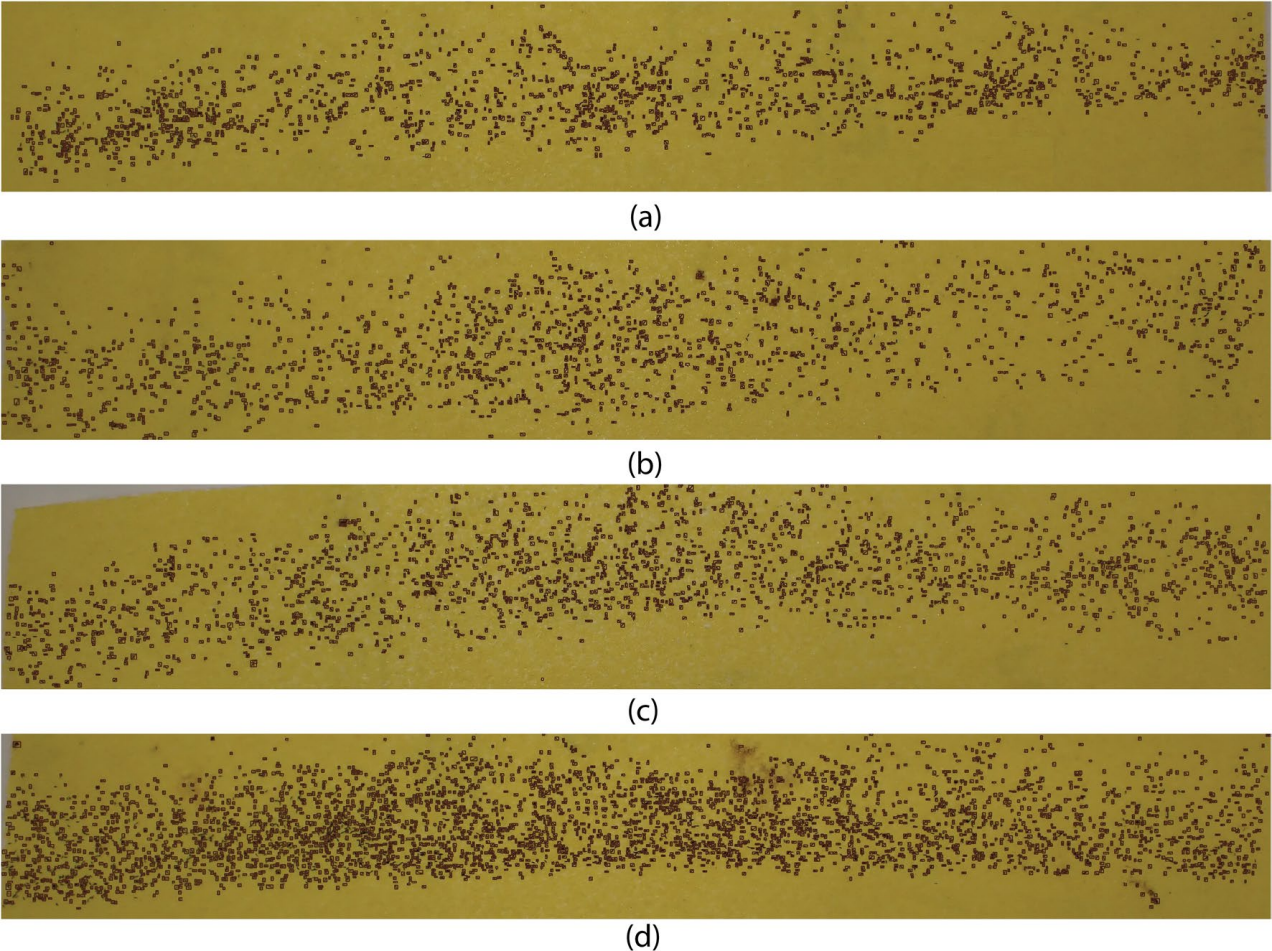
EggCountAI results based on varying density of macro images of *Ae. aegypti* eggs, with images showing **a** 1575, **b** 1724, **c** 2112, and **d** 3344 eggs detected

Figure 5a–d presents a combined image of four macro images out of the 10 processed images, where rectangles outline the detected eggs. The figure shows that EggCountAI has successfully detected most of the eggs, including the eggs as part of clusters.

## Comparative analysis of accuracy in EggCountAI, ICount, and MECVision

To further evaluate the performance of EggCountAI, we conducted a comparative analysis by comparing the results of EggCountAI with the results obtained from ICount and MECVision. The comparison was based on the overall percentage accuracy for the micro and macro images, as discussed below.

### Micro images

**Fig. 6:**
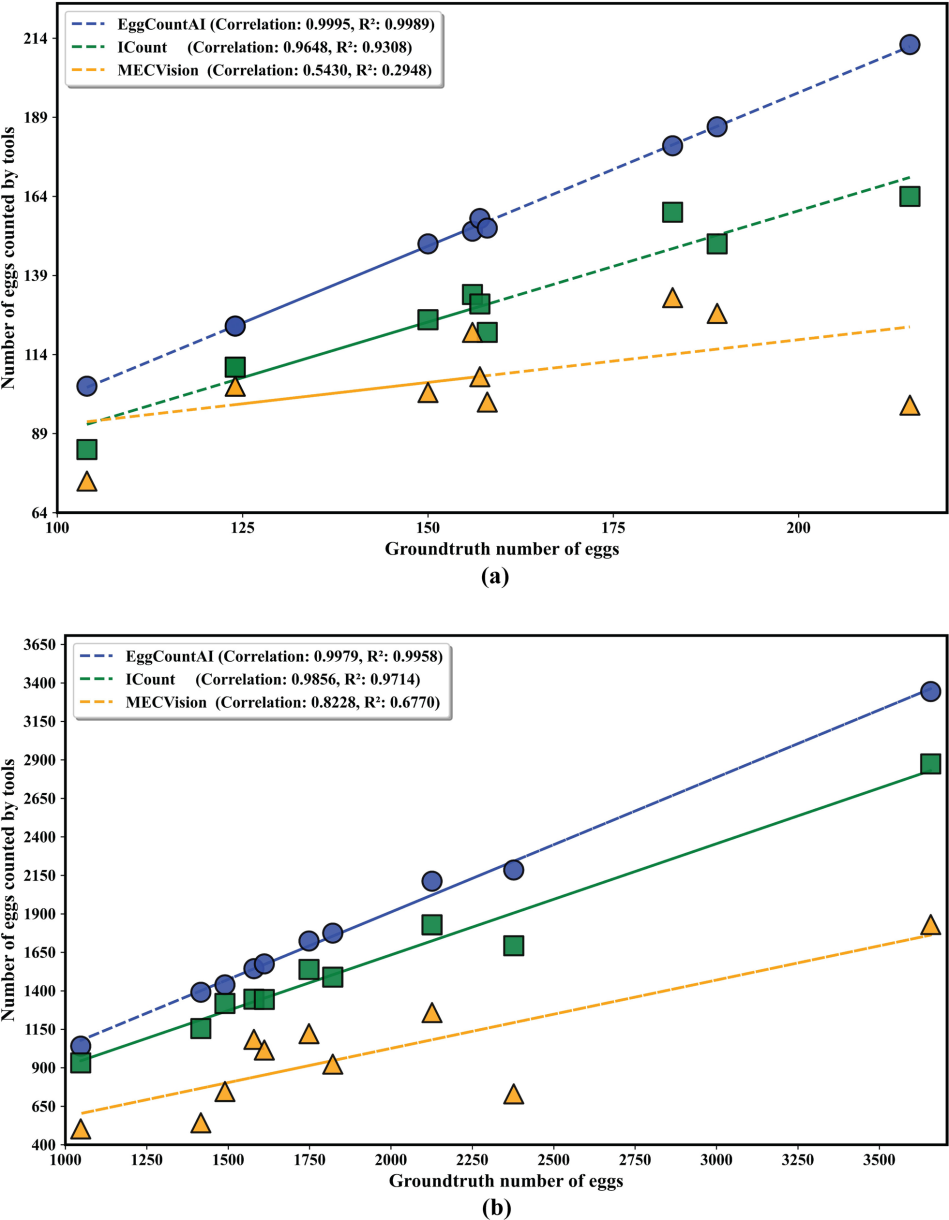
Correlation curves based on linear regression for comparative analysis of **a** micro images and **b** macro images

The previously used 10 micro images were run through the ICount and MECVision and compared with EggCountAI (Table 3). In terms of percentage accuracy, EggCountAI significantly outperformed both ICount and MECVision, with overall accuracy of 98.88%, versus 81.71% and 68.01%, respectively. In addition, EggCountAI demonstrated superiority in other statistical parameters, achieving mean absolute error of 1.90 eggs, showcasing highly precise estimations. Moreover, the predicted values and actual counts exhibited a strong relationship, with a correlation and *R*-squared value of 0.99 (Fig. 6a).

**Table 3:** Comparative analysis of EggCountAI, ICount, and MECVision based on micro images of *Ae. aegypti* eggs

Image	Ground truth	EggCountAI	ICount	MECVision
1	189	186	149	127
2	156	153	133	121
3	157	157	130	107
4	183	180	159	132
5	158	154	121	99
6	177	177	143	133
7	124	123	110	104
8	215	212	164	98
9	104	104	84	74
10	150	149	125	102
Overall accuracy %		98.88%	81.71%	68.01%
Mean absolute error		1.90	27.0	52.60
Correlation		0.99	0.96	0.54
*R*-squared		0.99	0.93	0.29

**Fig. 7:**
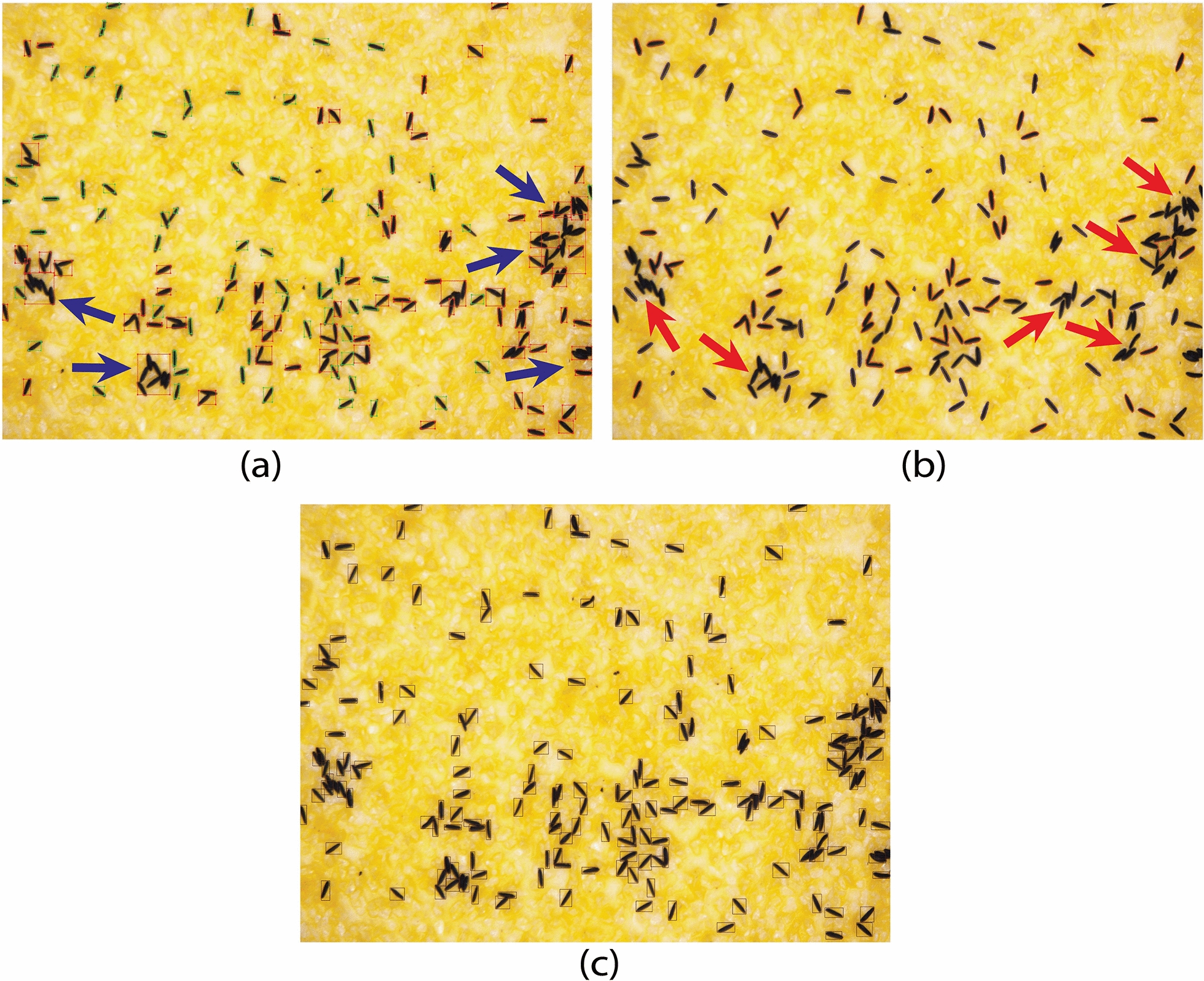
Comparative analysis of ICount, MECVision, and EggCountAI based on micro images of *Ae. aegypti* eggs. **a** A total of 149 eggs were counted by ICount. Blue arrows show the areas where eggs were overlapping or close to the neighbouring eggs. **b** A total of 127 eggs were counted by MECVision. Red arrows show the areas where the eggs were not counted. **c** A total of 186 eggs were counted by EggCountAI

When analysing micro images, results show that the accuracy of the ICount software decreased where eggs were overlapping or close to the neighbouring eggs (Fig. 7a), while the MECVision software even missed some single eggs as well as eggs in clusters (Fig. 7b). However, EggCountAI was able to successfully detect most of the eggs irrespective of their closeness and overlapping (Fig. 7c).

### Macro images

For comparison based on macro images, the macro images were processed through ICount and MECVision and compared with EggCountAI results (Table 4). EggCountAI again demonstrated superior performance relative to the other two, with overall accuracy of 96.06%. The overall accuracy of ICount and MECVision was 82.22% and 51.71%, respectively. In macro image analysis, EggCountAI continued its remarkable accuracy in other statistical parameters. It achieved mean absolute error of 74.30 eggs, providing reliable counts for macro images. Similar to micro images, the correlation and *R*-squared value of 0.99 indicated a strong relationship between predicted values and actual counts (Fig. 6b).

**Table 4:** Comparative analysis of EggCountAI, ICount, and MECVision based on macro images of *Ae. aegypti* eggs

Image	Ground truth	EggCountAI	ICount	MECVision
1	2377	2185	1693	731
2	1579	1544	1346	1085
3	1821	1775	1489	924
4	1416	1391	1155	543
5	1490	1439	1318	746
6	2126	2112	1829	1259
7	1748	1724	1539	1122
8	1047	1041	932	503
9	3658	3344	2874	1831
10	1611	1575	1344	1016
Overall accuracy %		96.06%	82.22%	51.71%
Mean absolute error		74.30	335.40	911.30
Correlation		0.99	0.98	0.82
*R*-squared		0.99	0.97	0.67

**Fig. 8:**
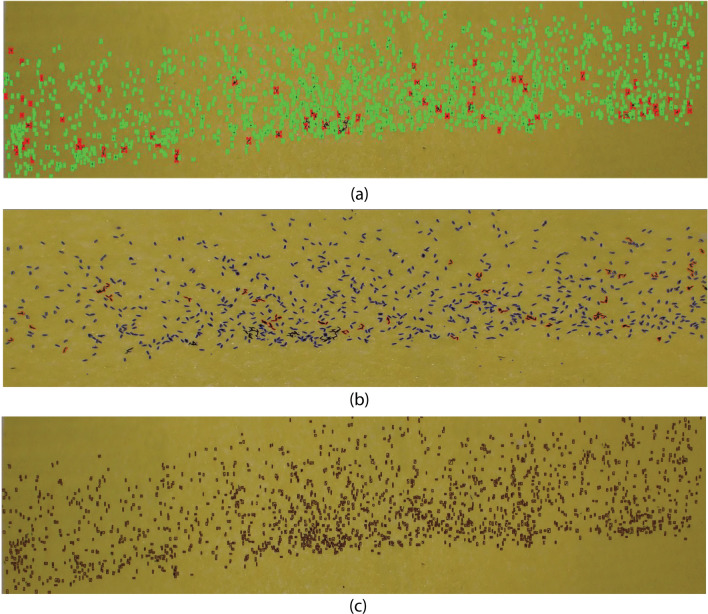
Comparative analysis of ICount, MECVision, and EggCountAI based on macro images of *Ae. aegypti* eggs. **a** A total of 1489 eggs were counted by ICount. **b** A total of 924 eggs were counted by MECVision. **c** A total of 1775 eggs were counted by EggCountAI

ICount analysis of macro images showed similar performance as in the case of micro images and missed eggs where they were overlapping or in the form of clusters (Fig. 8a). In the case of MECVision, along with missing a few single eggs and clusters of eggs, MECVision also cropped the macro input image in a small part (Fig. 8b), demonstrating that it might have some size limitation for the input image. If that is the case, it is tedious to crop the egg strip image in small chunks and then process it. Also, it was not confirmed whether it considers the whole input image for the counting or only the small part that it shows during processing. In contrast, regardless of egg proximity and overlapping, EggCountAI demonstrated a high success rate in detecting most of the eggs (Fig. 8c).

## Discussion

Vector control remains a vital strategy in the battle against mosquito-borne diseases. Information about fecundity is integral to effective mosquito management strategies. It can provide valuable data for surveillance and monitoring programmes [37], identifying areas with high mosquito populations [38] and potential breeding sites [39], and assessing the effectiveness of vector control interventions [40]. Fecundity information can also be used to assess physical measurements and fitness, as it has shown a strong correlation with pupal mass and wing size [41].

Manual counting of mosquito eggs is a laborious and time-consuming task. The use of automatic tools for egg counting offers numerous benefits, including reduced counting time, the ability to process more images, and avoidance of human errors. We have developed EggCountAI, which can easily count thousands of *Ae. aegypti* eggs, and with high accuracy of 98.88% in the case of micro images and 96.06% in the case of macro images. Additionally, EggCountAI has the potential to extend its high-accuracy counting to *Aedes albopictus* eggs, considering the importance of monitoring this invasive species in various regions.

Other egg-counting software such as ICount [24] and MECVision [23] require manual processing to upload individual images and for adjusting the input parameters for each image separately, as the size of the eggs and size of the clusters of eggs can vary in each image. Hence, separate adjustment of minimum egg size, maximum egg size, and maximum cluster size is needed. The advantage of EggCountAI is that it does not require adjustment of input parameters for each image separately, as it intelligently performs egg detection based on a CNN. Additionally, image uploading is very simple and does not require feeding each image separately, enabling the processing of a large number of images within a folder in a single operation without any supervision.

Oviposition traps (OVTs) have emerged as a crucial strategic monitoring instrument, owing to their greater sensitivity than that of larval or adult monitoring in *Aedes* control [42], and have recently gained widespread recognition for their efficacy in monitoring and reducing *Aedes* environmental populations [43]. Various tools have been used to count eggs from data acquired through OVTs [21, 44]. However, the number of eggs in each testing data sample was less than 500. Counting and recording eggs from the OVTs can pose significant challenges, particularly when manual counting is infeasible due to the sheer volume of eggs and their intricate distribution on the oviposition substrate. To address these issues, the integration of EggCountAI holds promise in overcoming difficulties associated with certain egg samples. While EggCountAI has exhibited high accuracy during evaluations under controlled lab conditions using images of sandpaper strips with no environmental influences, it is important to note that in endemic areas, egg samples can be considerably more complex, often featuring multiple layers of eggs on the substrate and vulnerability to environmental factors such as dust particles. To enhance detection accuracy in such areas, the choice of the substrate becomes crucial, as it should provide sufficient contrast between the substrate and eggs. Additionally, deploying OVTs for limited durations can help mitigate the formation of multiple egg layers. To minimise the impact of the open environment, custom-designed OVTs can be developed to exhibit greater resistance towards various environmental factors, further refining the monitoring and control of *Aedes* populations.

While EggCountAI can accurately count eggs within an image, it does have a limitation: it may cut off eggs located at the boundary of patches when creating a grid of rows and columns to analyse the image, which could result in eggs not being counted or counted multiple times during processing. A possible solution to this problem is to overlap across the boundary of patches. However, the inclusion of an overlap resulted in poor performance and was therefore excluded. Alternatively, the ability to use filtration parameter settings is helpful in reducing the impact of this problem. For instance, if the filtration parameter value is set to 3.0 and the grid patch cuts small parts of an egg across neighbouring patches that are three or more times smaller than the average size of the eggs, that would be filtered out during processing. In the case of multiple counting of a single egg, it can only be counted multiple times if it cuts the significant portions of the egg across neighbouring patches that fall out of the adjusted filtration value.

## Conclusion

The fitness traits of mosquitoes, including flight and egg-laying ability, are among the factors influencing disease spread. Current egg-counting tools require separate processing for each image and struggle with accuracy for clustered or overlapping eggs. Here we have presented EggCountAI, a free automatic artificial intelligence (AI)-based *Aedes* mosquito egg-counting tool. Compared with currently available software, EggCountAI performed remarkably well, with significantly increased accuracy and the ability to discern more complex samples when processing either micro or macro images. EggCountAI surpassed both ICount and MECVision in performance, achieving overall accuracy of 98.88% for micro images and an impressive 96.06% accuracy for macro images. In addition to its superior performance, EggCountAI offers advantages including the lack of need to adjust input parameters for each image individually, the batch processing of images, and effective filtration of undesired impurities.

EggCountAI has the potential to revolutionise mosquito research and disease control strategies. By accurately detecting and counting mosquito eggs, EggCountAI can help to identify critical egg-laying sites and strategically place OVTs, leading to a deeper understanding of mosquito behaviour and fitness traits. Embracing AI in mosquito research improves vector-borne disease modelling and enhances our understanding of pathogen transmission, leading to more effective disease prevention and proactive public health measures for a healthier and safer future.

This deep neural network-based egg-counting tool is one step towards employing AI in studying mosquito behavioural and fitness traits. Continuously developing and exploring AI-based tools can significantly enhance disease control and prevention strategies.

## Data Availability

The EggCountAI exe file, installation instructions, and test images are available for free download at the following link: https://drive.google.com/drive/folders/1SdICI1nNeVPuJtdgSUfOol46zoq4LYIj?usp=share_link.

## Abbreviations

RCNN: Region-based Convolutional Neural Network
LLINs: Long-lasting insecticidal nets
IRS: Indoor residual spraying
NMS: Non-maximum suppression
BSL-3: Biosafety level 3
ACDP: Australian Centre for Disease Preparedness
RO: Reverse osmosis
RPN: Region proposal network
